# Otorhinolaryngologic Manifestations of Tuberculosis: A Comprehensive Review of Clinical and Diagnostic Challenges

**DOI:** 10.7759/cureus.64586

**Published:** 2024-07-15

**Authors:** Jasleen Kaur, Prasad T Deshmukh, Sagar S Gaurkar

**Affiliations:** 1 Otolaryngology - Head and Neck Surgery, Jawaharlal Nehru Medical College, Datta Meghe Institute of Higher Education and Research, Wardha, IND

**Keywords:** multidisciplinary treatment, public health, antitubercular therapy, diagnosis, otorhinolaryngologic manifestations, tuberculosis

## Abstract

Tuberculosis (TB) is a significant global health issue, predominantly affecting the lungs but also capable of involving the otorhinolaryngologic (ear, nose, and throat) regions. This comprehensive review explores the epidemiology, pathophysiology, clinical presentation, diagnostic challenges, management strategies, and public health implications of otorhinolaryngologic TB. The disease's diverse clinical manifestations, such as chronic ear discharge, nasal obstruction, and hoarseness, often mimic other common conditions, complicating diagnosis and delaying treatment. Diagnostic confirmation requires a combination of clinical assessment, laboratory tests, and imaging techniques, each with inherent limitations. Effective management necessitates a multidisciplinary approach, integrating medical and surgical interventions tailored to individual patient needs. Potential complications, including airway obstruction and hearing loss, highlight the importance of timely and appropriate treatment. The review underscores the critical role of public health measures in TB control. It also identifies emerging trends in diagnosis and treatment, emphasizing the need for ongoing research to improve patient outcomes and contribute to the global effort to control and eventually eradicate TB. This review aims to give healthcare providers a deeper understanding of otorhinolaryngologic TB, enhancing diagnostic and therapeutic approaches and improving patient care.

## Introduction and background

Tuberculosis (TB) is a chronic infectious disease caused by *Mycobacterium tuberculosis *(M. tb). Although it predominantly affects the lungs, TB can also impact other body parts, including the otorhinolaryngologic (ear, nose, and throat) regions. TB remains a major public health issue, particularly in developing countries [1]. Despite the availability of effective treatments, the disease persists due to factors such as delayed diagnosis, inadequate treatment adherence, and the emergence of multidrug-resistant TB (MDR-TB). TB spreads from person to person through airborne droplets released when an infected individual coughs, sneezes, or speaks. The global burden of TB is staggering, with the World Health Organization (WHO) estimating approximately 10 million new cases and 1.4 million deaths in 2019. The disease is especially prevalent in regions with high rates of HIV, malnutrition, and poverty, where access to healthcare is often limited [2].

Otorhinolaryngologic manifestations of TB, although less common than pulmonary TB, are significant due to their potential for severe complications and diagnostic challenges. TB can affect various structures within the ear, nose, and throat, leading to various clinical presentations [3]. These manifestations often mimic other common otorhinolaryngologic conditions, complicating the diagnosis and delaying appropriate treatment. The involvement of otorhinolaryngologic structures in TB can lead to persistent symptoms such as chronic ear discharge, nasal obstruction, hoarseness, and non-healing ulcers. These symptoms can significantly impact a patient's quality of life and may lead to complications such as airway obstruction or hearing loss if not promptly and adequately treated [4].

This comprehensive review's primary objectives are to examine TB's epidemiology, provide an overview of the global burden, incidence, and prevalence trends, and focus on otorhinolaryngologic manifestations. It is essential to understand the pathophysiology of TB, exploring the microbiology of M. tb, its mechanisms of infection, and the spread of the disease to otorhinolaryngologic structures. This review also aims to describe the clinical presentation, outlining the common symptoms and signs of otorhinolaryngologic TB and emphasizing the variety and complexity of its manifestations. Addressing diagnostic challenges is another key objective, as well as discussing the difficulties in clinical diagnosis versus laboratory confirmation, the role and limitations of imaging techniques, and the challenges in differential diagnosis. Reviewing management strategies is crucial, detailing the medical management of TB, including antitubercular therapy, the indications and outcomes of surgical interventions, and the importance of a multidisciplinary approach to treatment. Identifying complications and prognosis highlights the potential complications of otorhinolaryngologic TB, as well as the long-term prognosis and follow-up considerations.

## Review

Epidemiology of TB

In 2022, an estimated 10.6 million incident cases of TB and 1.3 million deaths due to TB were reported globally [5]. The majority of TB cases and deaths occur in low- and middle-income countries, particularly in Asia and Africa [1,5,6]. India and China together account for 38% of the global TB burden. Approximately 90% of TB cases occur in adults, with the highest incidence rates among the most economically productive age groups. TB is a leading cause of death from infectious diseases worldwide, although mortality rates have declined by 42% since 1990 [1,5,6]. The global TB incidence rate has gradually declined by about 1.3% per year since 2002. However, the absolute number of global TB cases has remained relatively stable, with an estimated 8.6 million new cases in 2012 [1]. TB prevalence has been declining globally since the early 1990s, primarily due to the scale-up of the DOTS (directly observed treatment, short-course) strategy [1]. Despite this progress, the WHO End TB Strategy goals of a 90% reduction in TB deaths and an 80% reduction in incidence by 2030 are not currently on track to be met. Major challenges include the continued high burden in parts of Asia and Africa, the rise of drug-resistant TB strains, and difficulties in early case detection and treatment [1,7].

Pathophysiology of TB

Microbiology of M. tb

TB is caused by the bacterium M. tb, which is transmitted by inhaling infectious aerosol droplets from patients with active TB. After inhalation, the TB bacteria travel to the lungs and are phagocytosed by alveolar macrophages. However, M. tb can evade destruction by the macrophages and multiply within them. The host's immune system then mounts a cell-mediated response, recruiting lymphocytes and other immune cells to the site of infection. This leads to the formation of granulomas, the hallmark of TB pathology. In most cases, the immune system can contain the infection within the granulomas, leading to a latent TB infection. However, in some cases, the granulomas break down, allowing the bacteria to replicate and cause active, symptomatic TB disease. Active TB can manifest in the lungs (pulmonary TB) or other organs (extrapulmonary TB). Extrapulmonary TB, including otorhinolaryngeal manifestations, is less common but can still occur [8-11]. M. tb is a slow-growing, aerobic, acid-fast bacillus with a complex, lipid-rich cell envelope that makes it relatively resistant to the host's immune defenses and certain antibiotics. The bacilli's antigenic cell wall components, including glycoproteins, phospholipids, and wax D, activate the host's innate immune response. However, M. tb has evolved mechanisms to evade and delay the adaptive immune response. The high oxygen tension in certain organs, such as the lungs, kidneys, and meninges, makes them more susceptible to M. tb infection and the development of active TB disease [9,10].

Mechanisms of Infection and Spread

M. tb is transmitted through inhaling infectious aerosol droplets from patients with active TB. The bacteria then travel to the lungs and are phagocytosed by alveolar macrophages [11]. However, M. tb can evade destruction by the macrophages and multiply within them. The bacteria have evolved various mechanisms to inhibit phagosome-lysosome fusion, block acidification of phagolysosomes, and resist killing by the host immune system [12]. M. tb can also infect and replicate within other cell types, such as pulmonary epithelial cells and macrophages. This allows the bacteria to establish infection and persist in diverse intracellular environments [13]. When the granulomas that form to contain the infection break down, M. tb can replicate and cause active, symptomatic TB disease. This can manifest as pulmonary TB or extrapulmonary TB, including otorhinolaryngeal manifestations [11]. M. tb induces necrosis of infected host cells rather than apoptosis, which helps the bacteria disseminate to new sites and infect other cells. CpnT, phthiocerol dimycocerosates (PDIM), and iron overload contribute to this necrotic cell death [13]. The ability of M. tb to evade the host's immune responses, survive in diverse intracellular niches, and induce cell death mechanisms that favor bacterial spread is key to its successful infection and transmission cycle [13].

Clinical Presentation of Otorhinolaryngologic TB

Ear: TB of the ear, although rare, typically affects the mastoid air cells and the middle ear. Patients may present with painless, profuse otorrhea characterized by persistent and copious ear discharge. Hearing loss is another common symptom, often resulting from chronic infection affecting the structures of the middle ear. Additionally, patients might experience tinnitus, described as a ringing or buzzing noise in one or both ears, which can be constant or intermittent. Vertigo, a sensation of spinning or loss of balance, may also occur. Although less common, some individuals might suffer from earache or pain in the ear [14].

Nose: Nasal TB is uncommon but can significantly impact the patient's quality of life. Symptoms typically include nasal obstruction, making it difficult to breathe through the nose due to blockage. Chronic nasal discharge, known as rhinorrhea, is another frequent complaint. The discharge may vary in consistency and can be clear, purulent, or blood-stained. These symptoms can often lead to misdiagnosis, as they resemble those of more common nasal conditions [15].

**Figure 1:**
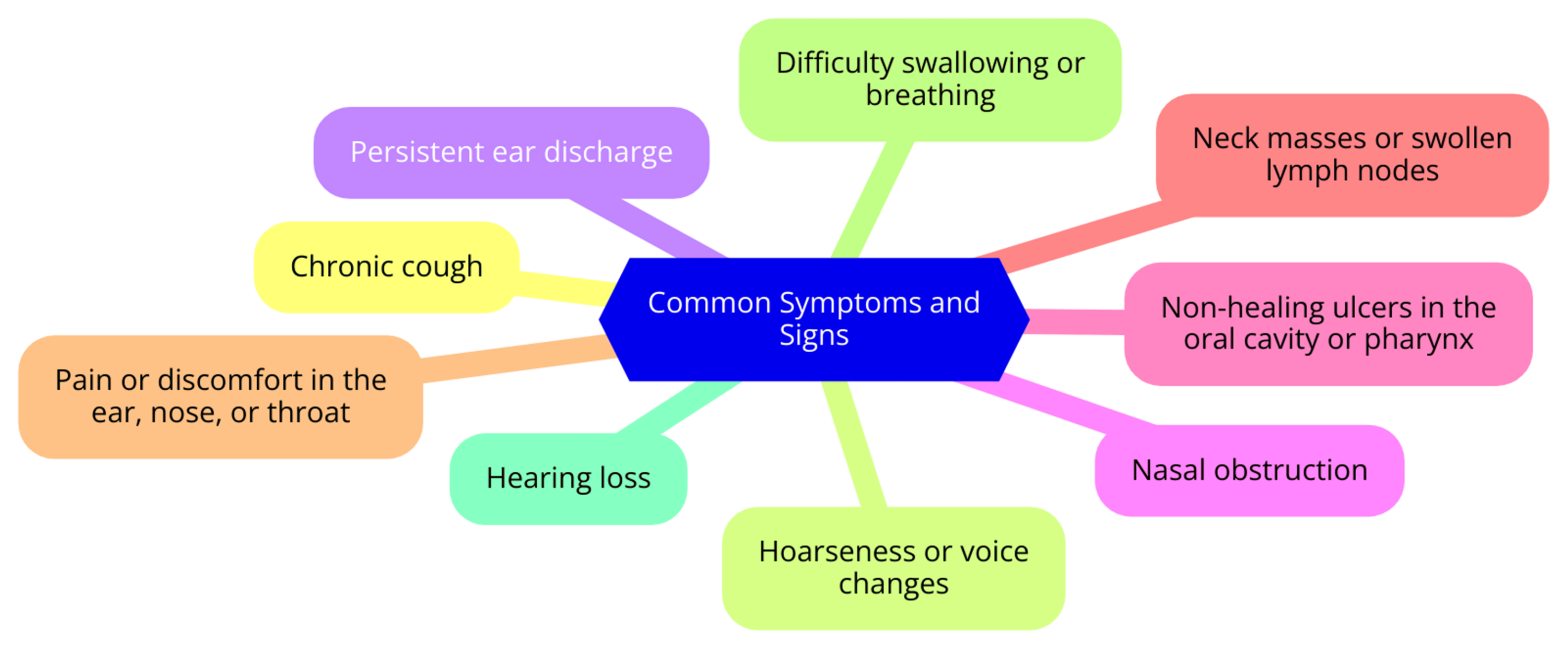
Common symptoms and signs of otorhinolaryngologic tuberculosis Image credit: Dr Jasleen Kaur

Larynx: Laryngeal TB is one of the more prevalent ENT manifestations of TB. It primarily affects the voice box and can lead to significant symptoms. Hoarseness, or changes in voice quality, including a raspy or hoarse voice, is a typical presentation. Patients may also experience odynophagia, which is painful swallowing, and dysphagia, indicating difficulty in swallowing. These symptoms often contribute to weight loss and a loss of appetite, as patients find eating increasingly challenging and unpleasant. The systemic symptoms of TB, including general malaise and weight loss, further complicate the clinical picture [16]. Common symptoms and signs are shown in Figure 1.

Diagnostic challenges in otorhinolaryngologic TB

Clinical Diagnosis Versus Laboratory Confirmation

Clinical diagnosis relies solely on symptoms and signs, while laboratory confirmation uses diagnostic tests to detect specific biomarkers, pathogens, or other indicators of disease [17]. Clinical diagnosis is often unreliable and lacks sensitivity and specificity, as many conditions have nonspecific symptoms that overlap between diseases [18]. Laboratory tests provide objective data to rule out or confirm suspected diagnoses. They are probabilistic, with sensitivity indicating the ability to detect true positives and specificity indicating the ability to exclude false positives [17,19]. Diagnostic tests have varying degrees of accuracy, reliability, and predictive value. Factors such as the purpose of testing (diagnosis, monitoring, screening), timing of sample collection, and test methodology impact the interpretation of results [17,19]. Clinical diagnosis and laboratory confirmation are complementary. Clinical suspicion guides test ordering, while test results inform and refine the clinical diagnosis. Integrating both is essential for accurate diagnosis and appropriate patient management [17,19].

Imaging Techniques: Role and Limitations

Imaging techniques such as computed tomography (CT) and magnetic resonance imaging (MRI) are invaluable in accurately visualizing the sites, patterns, and extent of TB lesions in the ENT region [20,21]. They alert clinicians to the possibility of TB and are particularly useful for imaging head and neck TB, providing detailed information about disease location and spread [22]. While CT and MRI can aid in diagnosis by showing the characteristic features of TB, they are not sufficient alone. Confirmation typically requires a combination of clinical suspicion, microbiological testing, and histopathological examination [3]. In cases where TB is strongly suspected but cultures are negative, polymerase chain reaction (PCR) testing of samples can be instrumental in detecting the disease, especially in lesions with low bacterial loads [22]. However, imaging has limitations, including its nonspecific appearance, which can resemble other conditions such as malignancies and chronic infections. Therefore, imaging findings must always be interpreted in the context of clinical presentation and other diagnostic tests. While CT and MRI play a crucial role in delineating the sites and extent of otorhinolaryngologic TB, they do not independently establish a definitive diagnosis. A multimodal approach integrating imaging with microbiology and histopathology is essential for accurately diagnosing this complex condition [20,21].

Challenges in Differential Diagnosis

TB of the otorhinolaryngeal region represents a relatively uncommon form of extrapulmonary TB, accounting for only 20% of all TB cases [23]. This rarity often results in TB not being initially considered in the differential diagnosis of various ENT symptoms, potentially leading to delayed diagnosis and management. The clinical presentation of otorhinolaryngologic TB is nonspecific, resembling symptoms seen in malignancies, chronic infections, and inflammatory disorders [8]. This similarity complicates differentiation based solely on clinical features, necessitating a thorough diagnostic workup to distinguish TB from other diseases. Unlike pulmonary TB, only 25%-30% of patients with otorhinolaryngeal TB also have concurrent pulmonary involvement [8]. The absence of pulmonary symptoms further complicates the diagnostic process and may delay appropriate treatment initiation. Otorhinolaryngeal TB lesions typically exhibit a paucibacillary nature, meaning they contain a low bacterial load. This characteristic reduces the sensitivity of diagnostic tests such as smear microscopy and culture, which rely on detecting the tubercle bacilli [8]. Obtaining adequate samples for microbiological and histopathological analysis from certain ENT subsites, such as the larynx and nasopharynx, can be challenging [23]. This difficulty in sampling, combined with the rarity, nonspecific clinical presentation, lack of pulmonary involvement, and paucibacillary nature of otorhinolaryngologic TB, collectively contribute to significant diagnostic challenges. A high index of suspicion is crucial to consider TB in the differential diagnosis of various ENT symptoms, facilitating timely diagnosis and appropriate management.

Management strategies

Medical Management: Antitubercular Therapy

The standard first-line treatment for drug-susceptible TB involves a six-month regimen comprising four drugs: isoniazid, rifampicin, pyrazinamide, and ethambutol for the initial two months, followed by isoniazid and rifampicin alone for the subsequent four months. In cases where the TB isolate is fully susceptible, ethambutol may be discontinued after the initial two-month phase [24]. For patients with isolated isoniazid resistance, the regimen is adjusted by discontinuing it and replacing it with rifampicin, pyrazinamide, and ethambutol for the entire six months [24]. Monitoring serum uric acid levels is essential for patients receiving pyrazinamide, and those on long-term ethambutol should undergo periodic visual acuity and color vision testing to detect potential side effects [24]. There is a burgeoning pipeline of new anti-TB drug candidates featuring novel mechanisms of action, facilitating the development of potential "pan-TB" regimens capable of treating drug-susceptible, MDR-TB, and extensively drug-resistant (XDR) TB [25]. Researchers are developing computational algorithms to prioritize promising drug combinations based on criteria such as drug penetration, efficacy against different mycobacterial subpopulations, and predicted synergistic effects. This approach aims to expedite the identification of regimens capable of shortening TB treatment durations [25]. Additionally, efforts are underway to repurpose existing drugs and explore novel drug delivery systems, such as nanoparticles, to enhance TB management strategies [26].

Surgical Interventions: Indications and Outcomes

Surgical resection is recommended for patients with infectious TB, particularly those with drug-resistant strains, after receiving at least six to eight months of appropriate anti-TB therapy [27]. This approach eliminates persistent, contagious cavities that do not respond adequately to medical treatment alone. Common surgical procedures include lobectomy, pneumonectomy, and thoracoplasty. Studies indicate a treatment success rate from 75% to 98% following surgery [27]. Surgery is also indicated for diagnosing and managing complex TB cases, such as tuberculous pleural empyema, extensively destroyed lungs, and tuberculomas [27]. However, the most severe TB cases, such as those involving bilateral cavities or extensively destroyed lungs, often cannot undergo surgery due to the high risks of operative and postoperative complications, including mortality [27]. Historically, surgical techniques such as pneumothorax induction, phrenic nerve crush, and thoracoplasty were employed in the pre-antibiotic era to collapse affected lungs and close cavities associated with TB [28]. Modern surgical approaches now primarily involve lung resection (lobectomy, pneumonectomy) and less invasive procedures such as bronchial artery embolization for managing massive hemoptysis [29]. Some centers have adopted new modifications, such as osteoplastic thoracoplasty, for treating complicated cavitary TB [28]. While surgery plays a crucial adjunctive role in managing TB, particularly in drug-resistant cases, determining the optimal timing and selecting appropriate patients remain challenging due to the significant morbidity associated with these procedures. Multidisciplinary care and ongoing research efforts are essential to enhance treatment outcomes and minimize risks for patients requiring surgical intervention.

Multidisciplinary Approach to Treatment

The WHO recommends the establishment of multidisciplinary "TB Consilium" teams comprising experts from various specialties such as pulmonology, infectious diseases, microbiology, and pediatrics to effectively manage complex cases of MDR-TB [30]. These teams offer specialized consultation and guidance on formulating optimal treatment regimens, managing adverse effects, and navigating other clinical challenges associated with MDR-TB care. In Uganda, a national DR-TB Consilium was established to specifically address challenging cases of drug-resistant TB complicated by comorbidities and drug toxicities. This initiative supported healthcare workers in peripheral facilities by assisting in designing tailored treatment plans for complex patients, underscoring the importance of enhancing the capacity of frontline providers [31]. Multidisciplinary teams involving physicians, nurses, epidemiologists, public health experts, laboratory specialists, and mental health professionals play pivotal roles in the comprehensive management of TB. They are crucial for ensuring accurate diagnosis, prescribing appropriate medications, managing treatment side effects, educating patients, tracking disease transmission, implementing targeted interventions, and addressing socio-behavioral factors that impact TB care and outcomes [32,33]. This collaborative approach helps optimize patient care, improve treatment adherence, and enhance overall TB control efforts globally.

Public health implications

Role of Public Health Measures in TB Control

Public health measures are pivotal in controlling the spread of TB. One of the primary interventions is early diagnosis and treatment. This involves maintaining a robust surveillance system to promptly report individuals with suspected or confirmed TB, ensuring access to necessary laboratory and radiological tests, and initiating treatment as soon as possible. Continuous case management throughout treatment is crucial for effective TB control [34]. Another critical public health measure is contact investigation and treating latent TB infection. This includes identifying, managing, and treating contacts and other individuals infected with M. tb to prevent the progression to active TB disease. Testing populations at high risk for latent TB infection and providing appropriate treatment are essential components of this strategy [34]. Infection control measures also play a vital role in TB control efforts. This involves implementing administrative, environmental, and personal protection measures to reduce the risk of TB transmission in healthcare settings and other congregate settings. Strengthening airborne infection control measures in healthcare facilities is particularly important [34]. Community engagement and advocacy are integral to a comprehensive public health approach to TB control. Engaging and mobilizing communities to participate in TB control efforts and utilizing communication strategies for social mobilization are essential for fostering a community committed to TB control and elimination. Finally, partnerships and collaboration are fundamental to achieving effective TB control and eventual elimination. Public health programs must collaborate closely with high-risk stakeholders and populations to leverage resources and maximize impact. Achieving TB elimination requires concerted efforts beyond the scope of public health programs alone [35].

Strategies for Early Detection and Prevention

Maintaining a high index of suspicion for TB among clinicians is crucial for the early detection of otorhinolaryngologic (ENT) manifestations of the disease. These ENT presentations often mimic more common conditions such as malignancies, chronic infections, and inflammatory disorders, posing significant diagnostic challenges. Establishing a diagnosis requires a comprehensive approach that integrates clinical assessment, mycobacterial culture, and histopathological examination, as no single test is definitive [8]. Newer rapid diagnostic tests, such as cartridge-based nucleic acid amplification test (CBNAAT), offer faster results than traditional culture methods and should be considered in the diagnostic process. Timely diagnosis is critical, as delays can lead to increased morbidity. Studies have highlighted varying diagnostic delays, with ear TB having the longest median time to diagnosis (170 days), followed by laryngeal TB (84 days) and cervical lymphadenitis (48.6 days), underscoring the urgency of early detection [36]. Preventively addressing underlying risk factors for TB, such as poverty, malnutrition, and immunodeficiency conditions, can mitigate the risk of extrapulmonary TB, including ENT manifestations. Strengthening TB control programs to ensure early detection and treatment of pulmonary TB is pivotal, as this can prevent the spread of the disease to other organs [37]. Enhancing access to and utilization of TB screening and diagnostic services, particularly in regions with high disease burden, is crucial for facilitating early case detection. Public awareness campaigns focusing on TB symptoms and the importance of seeking prompt medical attention can also help reduce delays in diagnosis and treatment, thereby improving outcomes [38].

## Conclusions

Otorhinolaryngologic manifestations of TB, although less common than pulmonary TB, pose significant clinical and diagnostic challenges due to their diverse presentations and potential for severe complications. Timely diagnosis and appropriate management are crucial for preventing long-term morbidity and improving patient outcomes. This review highlights the necessity of a multidisciplinary approach to treatment, integrating medical and surgical interventions, to address the complex needs of patients with otorhinolaryngologic TB. Public health measures, including early detection, prevention strategies, and improved healthcare access, are vital in controlling the spread of TB. Continued research and innovation in diagnostic and therapeutic techniques are essential to enhancing our understanding and management of this disease. By increasing awareness and knowledge among healthcare providers, we can better address the diagnostic dilemmas and therapeutic challenges associated with otorhinolaryngologic TB, ultimately contributing to the global effort to control and eradicate TB.
